# Droplet duos on water display pairing, autonomous motion, and periodic eruption

**DOI:** 10.1038/s41598-023-39094-6

**Published:** 2023-07-31

**Authors:** Yutaka Sumino, Ryo Yamashita, Kazuki Miyaji, Hiroaki Ishikawa, Maho Otani, Daigo Yamamoto, Erika Okita, Yasunao Okamoto, Marie Pierre Krafft, Kenichi Yoshikawa, Akihisa Shioi

**Affiliations:** 1 Department of Applied Physics, Tokyo University of Science, 6-3-1 Niijuku, Katsushika, Tokyo, 125-8585 Japan; 2 Department of Chemical Engineering and Materials Science, Doshisha University, 1-3 Tatara Miyakodani, Kyotanabe, Kyoto 610-0321 Japan; 3 Department of Physics, Chiba University, 1-33 Yayoi-cho, Inage-ku, Chiba, 263-8522 Japan; 4 Department of Chemical Engineering, Osaka Metropolitan University, Gakuen-cho, Naka-ku, Sakai, Osaka 599-8531 Japan; 5 Research Center for Membrane and Film Technology, Kobe University, Kobe, 657-8501 Japan; 6 Institut Charles Sadron (CNRS), University of Strasbourg, 23 rue du Loess, 67034 Strasbourg, France; 7 Faculty of Life and Medical Sciences, Doshisha University, Kyoto, 610-0394 Japan; 8 Center for Integrative Medicine and Physics, Institute for Advanced Study, Kyoto University, Kyoto, 606-8501 Japan

**Keywords:** Surface chemistry, Chemical physics, Nonlinear phenomena

## Abstract

Under non-equilibrium conditions, liquid droplets dynamically couple with their milieu through the continuous flux of matter and energy, forming active systems capable of self-organizing functions reminiscent of those of living organisms. Among the various dynamic behaviors demonstrated by cells, the pairing of heterogeneous cell units is necessary to enable collective activity and cell fusion (to reprogram somatic cells). Furthermore, the cyclic occurrence of eruptive events such as necroptosis or explosive cell lysis is necessary to maintain cell functions. However, unlike the self-propulsion behavior of cells, cyclic cellular behavior involving pairing and eruption has not been successfully modeled using artificial systems. Here, we show that a simple droplet system based on quasi-immiscible hydrophobic oils (perfluorodecalin and decane) deposited on water, mimics such complex cellular dynamics. Perfluorodecalin and decane droplet duos form autonomously moving Janus or coaxial structures, depending on their volumes. Notably, the system with a coaxial structure demonstrates cyclic behavior, alternating between autonomous motion and eruption. Despite their complexity, the dynamic behaviors of the system are consistently explained in terms of the spreading properties of perfluorodecalin/decane duplex interfacial films.

Liquid droplets under nonequilibrium conditions are systems that mimic biological dynamics^1–8^. For example, the growth of droplets fueled by chemical reactions triggers their division into smaller daughter droplets^9^. The pairing of heterogeneous elements is another biologically relevant dynamics^10,11^. Furthermore, the cyclic occurrence of eruptive events such as necroptosis^12^ or explosive cell lysis^13,14^ is necessary to maintain cell functions. Unlike the self-propulsion behavior of cells^15–18^, cyclic cellular behavior involving pairing and eruption has not been successfully modeled using artificial systems. Recent studies show that the pairing dynamics of heterogeneous elements are artificially reproduced^19–22^. Indeed, the pairing of different cell types, common in biology, is necessary to enable collective activity^10^. Such pairing leads to cell fusion, which is an important step in embryogenesis and tissue development, and is used to generate antibody-secreting hybridomas or to reprogram somatic cells^11^. Extensive theoretical studies have been performed to investigate dynamics with non-reciprocal interaction^23, 24^.

In biomimetic investigations of the dynamics of active elements, especially those related to biological cells, programmed repetitive cyclic eruptive events have been overlooked. Eruptive events commonly occur in cell dynamics; for instance, necroptosis, a category of regulated cell death triggered by an external trauma, is regarded as a partially programmed event of cellular explosion^12^. In addition, explosive breaking down of membrane cell (lysis) has been identified as a mechanism in the biogenesis of bacterial membrane vesicles and biofilms,^13^ as well as in the destruction of cells by bacteria^14^. However, unlike the self-propulsion function, which has been described using various artificial systems^15–18^, to the best of our knowledge, no artificial system has been reported that mimics cyclic cellular behavior involving pairing and eruption. We developed a simple model consisting of a pair of droplets of quasi-immiscible hydrophobic oils (a perfluorocarbon (perfluorodecalin, PFD) and a hydrocarbon (decane)) deposited on the surface of water.

Interfacial phenomena are often driven by the effect of evaporation. Indeed, apart from the rich literature on the dynamics of droplet motion already mentioned, coffee-ring effect creates fascinating dynamics at the surface of the solution^25, 26^. High evaporation rate results in a nonequilibrium phenomenon with mass flux, which includes non-trivial droplets dynamics. Perfluorocarbon droplets exhibit specific behaviors owing, in particular, to their high vapor pressure relative to their molecular weight and to their quasi-immiscibility with both water and hydrocarbons^27, 28^. For example, in has been reported that perfluorocarbon droplets spontaneously self-organize on water to form hexagonal arrays with unique cyclic collective contraction and expansion behavior^29^. Recently, chemotactic signaling between perfluorocarbon and hydrocarbon droplets in surfactant solutions was shown to produce predator–prey-like chasing interactions^20^.

Here, we show that the developed system consisting of two droplets, one decane and one perfluorodecalin, deposited on the surface of water demonstrates unprecedented dynamic behavior. The experimental setup is illustrated in Fig. 1a. A PFD droplet is deposited on water before the deposition of an adjacent decane droplet (note that the order of deposition of the droplets does not affect the behavior of the system). The two droplets make contact spontaneously due to capillary interactions^30, 31^ and pair to form two distinct types of structures, depending on both their absolute and relative volumes. Smaller droplets, which are less deformable, remain attached, forming a Janus structure. Whereas larger droplets form a coaxial structure, i.e., a central PFD droplet surrounded by a decane ring. Interestingly, the coaxial structure thus formed undergoes eruptive episodes or periodic destruction and reconstruction (Fig. 1b and Supplementary movie 1). The coaxial structure forms approximately 160 s after the deposition of the droplets. A unique dynamic behavior, reminiscent of a volcanic eruption, is subsequently observed in which PFD and decane are forcefully repelled (as exemplified in Fig. 1b, 310 s). A transient state, in which decane forms lenses around PFD (777 s), leads to a second eruption (836 s). After 1602 s, the coaxial structure regenerates itself and another eruptive episode commences. This cycle repeats itself several times (see Supplementary movie 1).

**Figure 1:**
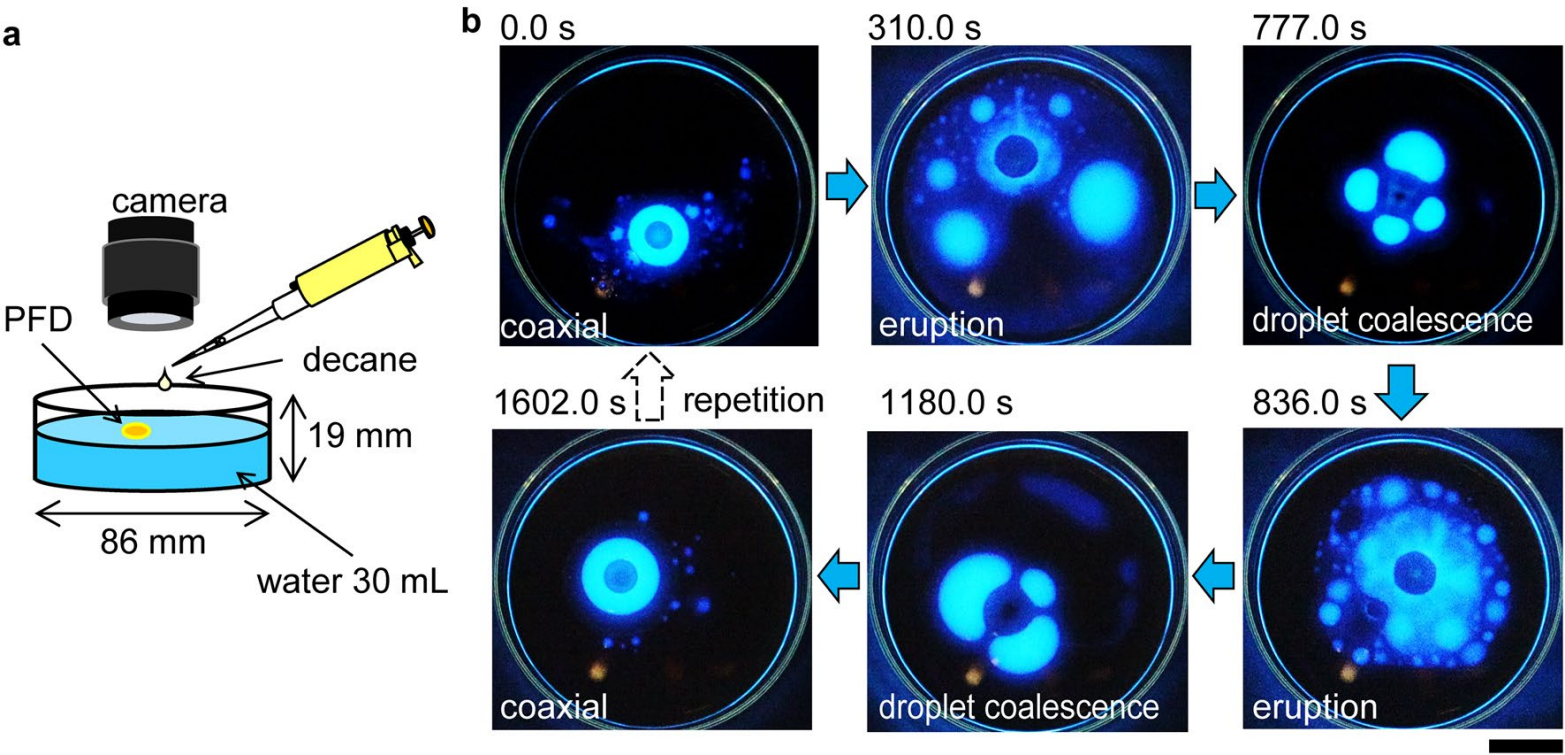
Dynamics and cyclic eruptive behavior of a droplet system. (**a**) Schematic representation of the experimental setup. (**b**) Fluorescence images depicting the typical cyclic dynamic behavior of the droplet system with a coaxial structure (a fluorescently labeled ring of decane surrounding a PFD core). The volumes of the PFD and decane droplets are both 150 µL. The top-left image was taken 156 s after depositing the droplets. The time elapsed since the top-left image was taken is shown in each snapshot. The scale bar corresponds to 20 mm.

To elucidate the unique dynamic behavior of the droplet system, we systematically investigated the effects of the absolute and relative volumes of the oils (10–200 µL) on the structure of the droplet system. The results are summarized in the phase diagram shown in Fig. 2a. In the yellow domain, a decane ring completely surrounds the PFD droplet, forming a coaxial structure. In the blue domain, decane and PFD droplets are attached, forming a Janus structure; reminiscent of Janus structures have been reported for other systems^19, 32, 33^. The generation of the Janus structure is attributable to the insufficiency of the volume of decane to form the coaxial shape: The droplet is expected to take a globular shape when the radius is less than the capillary length, approximately 4.3 mm that is calculated from $\sqrt{\gamma /g\Delta \rho };$ typical values of the water/decane interfacial tension ($\gamma$=50 mN/m), the difference in densities ($\Delta \rho =$ 0.27 g/cm^3^) and gravitational acceleration ($g=$ 9.8 m/s^2^). Along the border between the Janus and coaxial structure domains in the phase diagram, the transition between the structures is gradual. Figure 2b shows the change in the fractions of Janus versus coaxial structures for droplets of equal volume (dotted diagonal line in the diagram of Fig. 2a). The structural transition occurs at droplet volumes of 50 to 100 µL. In this transition region, the Janus and coaxial structures may be regarded as bistable.

**Figure 2:**
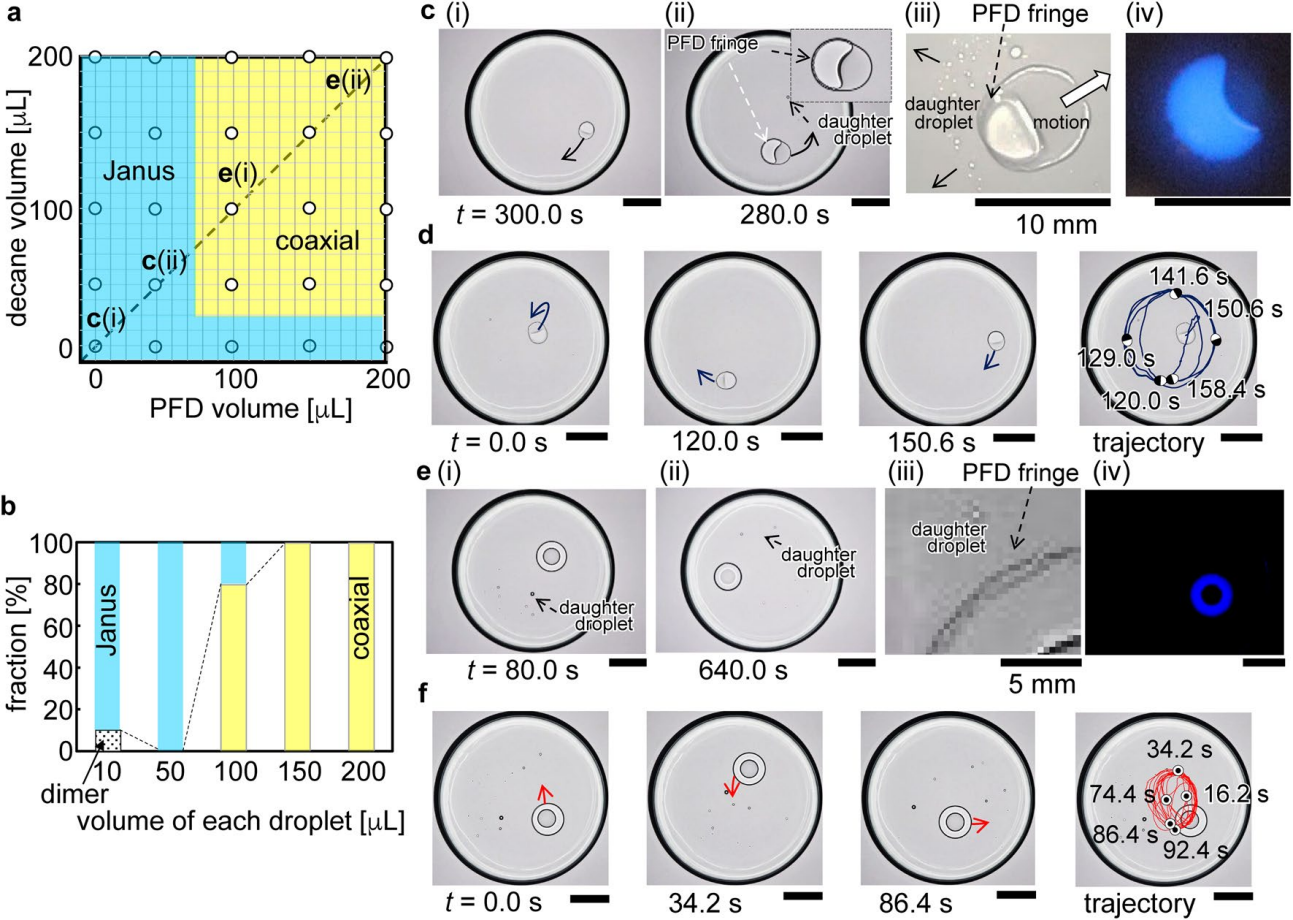
Change in the structure of the droplet system with change in droplet volumes and dynamic system behavior. (**a**) Phase diagram showing domains that correspond to each type of structure: Janus (blue) and coaxial (yellow). The circles represent the experimental volumes investigated. (**b**) Fraction of each type of structure formed in systems featuring droplets with equal volumes, corresponding to data points along the dotted diagonal line in (**a**). These fractions were determined from 10 repeated observations. For a 10 μL droplet, we observed a dimer structure, which resembles to a Janus structure. The dimer structure is characterized by a finite gap between droplets that do not touch directly. This dimer structure appears frequently when a small amount of surface-active agents is used (Fig. 4c(ii)). (**c**), (**e**), Shadowgraphs of systems denoted by **c(i)** and **c(ii)** (Janus), and **e(i)** and **e(ii)** (coaxial) in **a**; **c(iii)** and **e(iii)** are enlarged bright-field images of Janus and coaxial structures, respectively. Fluorescence images of Janus and coaxial structures, where only decane is fluorescently labeled, are shown in **c(iv)** and **e(iv)**, respectively. The coaxial structure is stable for at least 30 min. (**d**), (**f**), Snapshot shadowgraphs and trajectories of droplet systems with (**d**) Janus (**c(i)**) and (**f**) coaxial (**e(i)**) structures. The decane and PFD droplet volumes are 10 µL (**c(i)** and** d**), 50 µL (**c(ii), c(iii)**, and **c(iv)**) 100 µL (**e(i)**, **e(iii)**, **e(iv)**, and **f**), and 200 µL (**e(ii)**). Scale bars correspond to 20 mm unless stated otherwise.

Next, we investigated the kinetics of the droplet systems. The kinetic behavior of the droplet system with a Janus structure is exemplified in Fig. 2c(i),c(ii),d, and Supplementary movie 2. The decane droplet is fluorescently labelled with perylene, which is only soluble in decane (Fig. 2c(iv)). Careful observation reveals the formation of some smaller PFD droplets, as well as a fringe of PFD that expands at the air/water/decane triple-phase contact line (Fig. 2c(ii),c(iii)). This fringe completely lines the triple-phase contact line and stabilizes the PFD droplet. The autonomous motion of the Janus structure subsequently commences, with the PFD droplet in front, during which daughter droplets are expelled from the triple-phase contact line around the decane droplet (Figs. 2c(ii),c(iii)). This autonomous motion follows a regular circular trajectory in which the distance between the Janus structure and sidewall of the Petri dish remains nearly constant (Fig. 2d and Supplementary movie 2). This autonomous motion continues until the PFD has completely evaporated.

The kinetic behavior of the droplet system with a coaxial structure is exemplified in Fig. 2e(i),e(ii),f, and Supplementary movie 3. The droplets form a transient Janus structure before the PFD droplet is gradually encircled by the decane droplet to form a concentric structure (Supplementary movie 3). As in the case of the Janus structure (Fig. 2e(iii)), a PFD fringe is present at the air/water/decane triple-phase contact line. Moreover, like the Janus structure, the coaxial structure moves spontaneously across the water surface, the motion is accompanied by the expulsion of daughter droplets from the triple-phase contact line (Fig. 2e(iii)). The PFD fringe and expulsion of daughter droplets are shown in greater detail in supplementary Fig. S1. Decane is identified as the outer ring by fluorescence imaging (Fig. 2e(iv)). The circular trajectory of the coaxial structure appears to be somewhat less regular (Fig. 2f) than that of the Janus structure (Fig. 2d). In addition, the system occasionally demonstrates a dynamic behavior, i.e., the disruption of the decane ring by a channel, as shown in Fig. S2. However, the channel eventually closes again and the system recovers its coaxial structure. This channel opening and closing behavior is repeated several times and relies on balance between the interfacial tensions of the different phases present. It is expected that the formation of a duplex film of decane and PFD plays the essential role to generate the specific Marangoni flow as interpreted in Fig. 3.

**Figure 3:**
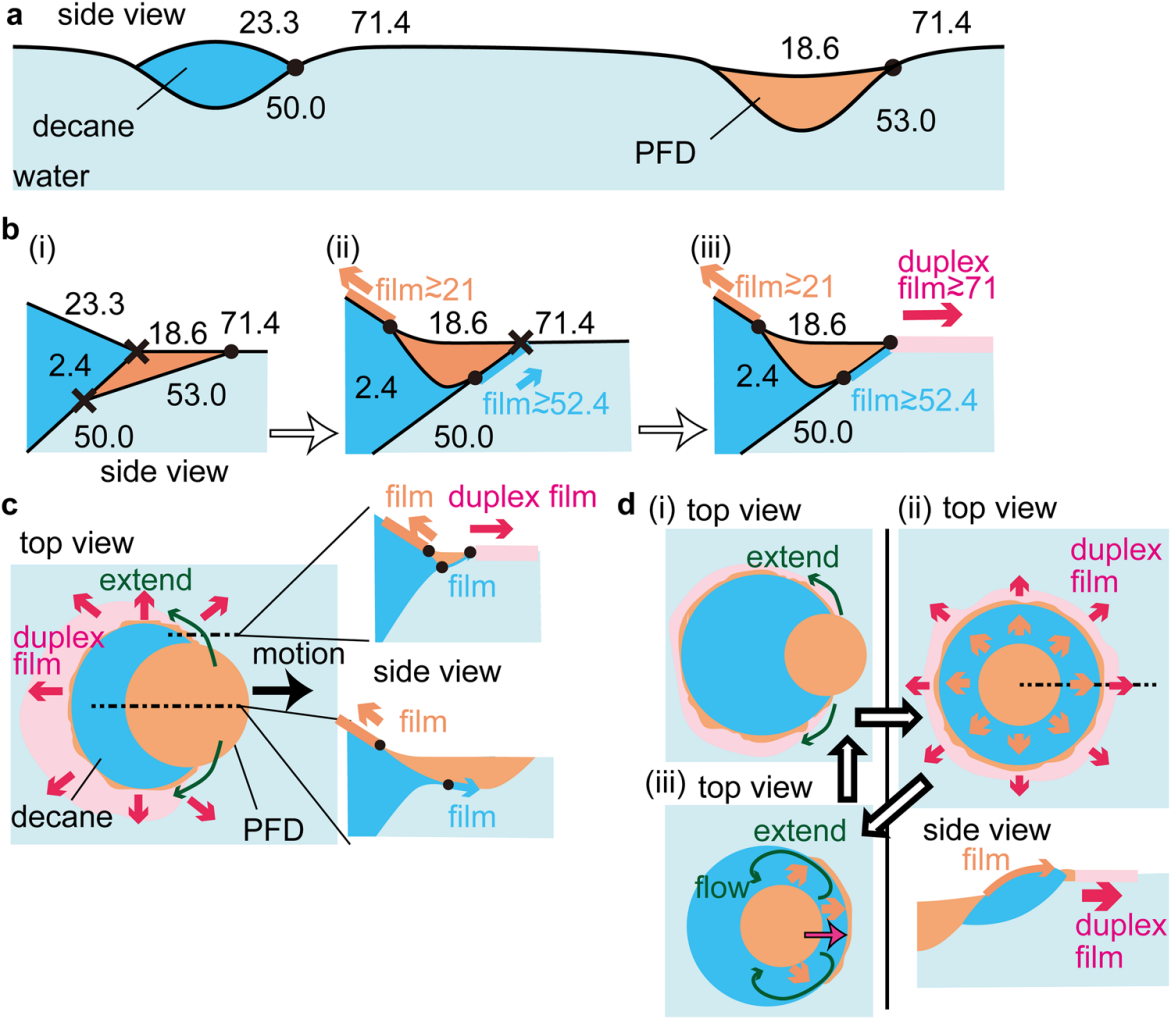
Schematic representation of triple-phase contact line and droplet system dynamics. (**a**), (**b**), Balance of the interfacial tensions (mN/m) at the triple-phase contact lines. (**a**) Decane and PFD droplets suspended at the air/water interface; light blue, white, bright blue, and orange regions correspond to water, air, decane, and PFD, respectively. (**b**) PFD droplets in contact with decane droplets; the black dots and crosses correspond to triple-phase contact lines that do or do not, respectively, satisfy the conditions of Neumann’s triangle. The light blue and orange arrows in **b(ii)** indicate the direction of film expansion and the corresponding values indicate the effective interfacial tensions of these films. The pink arrow and corresponding value in **b(iii)** indicate the direction of expansion of the decane/PFD duplex film and its effective interfacial tension, respectively. (**c**) Droplet system with a Janus structure and flux of PFD away from the structure. As shown by the green arrows, PFD expands at the air/water/decane triple-phase contact line, forming a fringe around the decane droplet. The duplex film extends from this PFD fringe along the air/water interface due to Marangoni flow. The droplet experiences a net force that causes its motion in the relative direction of the PFD droplet (black arrow) while expelling daughter droplets composed of duplex film. Side views indicate the directions of expansion of the PFD and duplex films. (**d**) Droplet system with a coaxial structure. At sufficiently high decane and PFD droplet volumes, the decane droplet engulfs the PFD droplet after the air/water/decane triple-phase contact line has been lined with a PFD fringe, as shown in **d(i)** and **d(ii)**. PFD at the center of the coaxial structure continuously flows to the fringe and air/water interface where daughter droplets composed of duplex film are expelled. When the supply of PFD to the fringe becomes insufficient, the latter disappears as shown in **d(iii)**. A flow then appears due to this PFD imbalance, causing the central PFD droplet to erupt from the coaxial structure. The coaxial structure is subsequently regenerated (**d(i)**). This process repeats itself.

Physicochemical studies of two immiscible sessile drops or liquid phases on a solid substrate have been studied^34–38^. In the present study, the dynamics of two immiscible liquid drops on the third liquid surface is investigated. To explain the above behaviors, we examined the tensions (or energies) at the six distinct interfaces between the four distinct phases present in the system. The notation of P_1_/P_2_/…/P_*n*_ was used to label the interfaces and films composed of phases 1 to *n*. Accordingly, P_1_/P_2_ represents the interface between phases 1 and 2, while P_1_/P_2_/P_3_ represents the film of phase 2 at the interface between phases 1 and 3. Table 1 lists the determined interfacial tensions between the various phases.

**Table 1:**
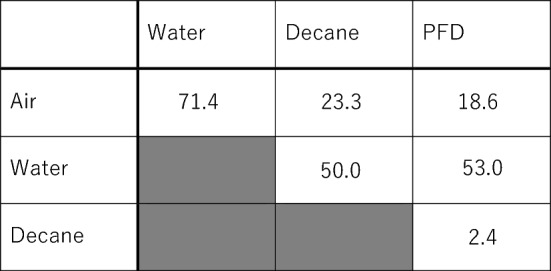
Interfacial tensions, mN/m, at room temperature and measured at equilibrium between the phases present in the quaternary system. The shaded regions indicate duplicated items.

These values were obtained for corresponding saturated phases; e.g., the interfacial water/decane tension is measured between decane-saturated water and water-saturated decane. The estimated deviation is ± 0.5 mN/m.

The effective interfacial tension of an air/PFD/decane/water system, calculated by simply adding the air/PFD, PFD/decane, and decane/water interfacial tensions, is 71.0 (= 18.6 + 2.4 + 50.0) mN/m, which is slightly less than the air/water interfacial tension, 71.4 mN/m (at room temperature). The effective interfacial tensions of an air/decane/PFD/water, air/decane/water, and air/PFD/water systems are comparatively high (78.7, 73.3, and 71.6 mN/m, respectively). Therefore, PFD and decane on water form a duplex film with air/PFD/decane/water layering. Once formed, this duplex film spontaneously spreads along the air/water interface. There are two other possible film-forming configurations: a PFD film on a decane surface (air/PFD/decane: 21.0 mN/m, which is less than the air/decane interfacial tension of 23.3 mN/m), and a decane film between PFD and water (PFD/decane/water: 52.4 mN/m, which is less than the PFD/water interfacial tension of 53.0 mN/m). The effective interfacial tensions of the air/PFD/decane/water and air/PFD/decane systems change as volatile PFD evaporates. Such a change in effective interfacial tension induces the spread, Marangoni flow, of the PFD/decane duplex film formed at the air/water interface and the PFD film formed at the air/decane interface.

The effective interfacial tensions of the two possible single component droplet systems, air/PFD/water (81.6 mN/m) and air/decane/water (73.3 mN/m), exceed the air/water interfacial tension, accounting for the formation of PFD and decane droplets, respectively, at the air/water interfaces of these systems. The spread of liquids along such interfaces can be described using the contact angles in two equilibrium states, namely, when the liquid totally wets the interface or only partially wets it, thus forming a lens. Neumann’s equation relates the interfacial tensions with the contact angles of droplets located at an interface. The transition between wetting stages is expressed by the spreading coefficient *S*, i.e., the difference in surface energy between partial and complete wetting.

$$S= {\gamma }_{1,n}-\sum_{i=1}^{n-1}{\gamma }_{i,i+1}$$

Here, ${\gamma }_{1, n}$ is the interfacial tension between phases 1 and *n*. When the spreading coefficient is positive, a film composed of 2/…/*n *− 1 phases spreads between phases 1 and *n* until the contact angle becomes zero and produces film structure. A negative spreading coefficient corresponds to partial wetting.

The spreading mechanism for the present system is summarized in Fig. 3, we consider the possible phase combinations near the triple-phase contact lines. As shown in Fig. 3a, both decane and PFD form lenses at the air/water interface (*S* is negative in both cases, − 1.9 and − 0.2 mN/m, respectively).

When decane and PFD droplets come into contact, three different triple-phase contact lines are formed, as shown in Fig. 3b(i). However, two of them (marked with crosses) do not satisfy the conditions of Neumann’s triangle. This implies that a PFD film is formed at the air/decane interface, and a decane film at the PFD/water interface (Fig. 3b(ii)). The PFD film easily evaporates, which leads to a continuous flow of PFD from the PFD droplet along the air/decane interface. This behavior is confirmed by an experiment in which a PFD droplet is deposited on decane and for which a strong and steady Marangoni flow is observed along the air/decane interface (Supplementary movie 4) as long as PFD is present. Furthermore, once the decane film at the PFD/water interface reaches the air/water interface, the air/PFD/decane triple-phase contact line becomes unstable, leading to the formation of a PFD/decane duplex film between the air and water phases (Fig. 3b(iii)). This duplex film can then spread at the interface. Due to the evaporation of the PFD present in the duplex film, the latter eventually only contains decane. This film is unstable at the air/water interface and therefore transforms into a decane lens. Here, also, the spatial gradient of the interfacial tension drives flow away from PFD/decane structure.

Marangoni forces are well known to cause various interfacial instability such as the interfacial turbulence^39^, tension oscillation^40^, movable contact line dynamics^41, 42^, self-propelling droplets^43, 44^ and particles^45^, and pattern-forming dynamics^46–48^. In addition, cloaked droplet, where a liquid film expands on an immiscible drop has been reported^49^. In the present study, the duplex film formation and the difference in the evaporation rate of each liquid produces the recursive droplet dynamics with translation, eruption, holing and pattern formation.

Let us now turn to the detailed interpretation of the experimental results. When relatively low volumes of decane and PFD are deposited on water in air (Fig. 3a), the balance of the interfacial tensions suggests that these phases partially wet the water and form lens-type droplets, which is consistent with the experimental results. Capillary interaction is responsible for the attraction between decane and PFD droplets until they eventually merge to form a Janus structure, as schematically represented in Fig. 3c. PFD spreads at the air/water/decane triple-phase contact line to form a fringe, as seen in Fig. 2c(iii); PFD flows across the decane surface from the PFD droplet to the fringe. However, the PFD supplied to the fringe does not develop into a PFD droplet because a PFD/decane duplex film spreads outward, along the air/water interface. It is noteworthy that decane also penetrates the PFD/water interface to form a film. The flow of the PFD/decane duplex film is supported by the continuous supply of decane. Overall, the evaporation of PFD creates a gradient in the effective interfacial tension, which is needed to maintain the Marangoni flows. The outward flow of the duplex film is not symmetric, it is weaker on the side of the PFD droplet due to the limited supply of decane. The Janus structure is therefore propelled in an asymmetric manner, expelling daughter droplets of decane from the duplex film extending from the fringe, as shown in Fig. 2c(iii).

Decane and PFD droplets of sufficient volume are deformable due to a lower surface to the volume ratio. Thus, when comparatively high volumes of decane and PFD are deposited on water, a PFD fringe lines the air/water/decane triple-phase contact line, as shown in Fig. 3d(i). Then, the decane droplet engulfs the PFD droplet to form a coaxial structure as schematically represented in Fig. 3d(ii) and observed in Fig. 2e. In this system, PFD from the centrally located PFD droplet continuously flows across the decane ring to the fringe, a duplex film is formed at the triple-phase contact line and spreads outward from the decane periphery along the air/water interface. This expansion of the duplex film is schematically represented in Fig. 3d(ii) and observed in Fig. 2e(iii). The duplex film is even more clearly observed in high-contrast bright-field and fluorescence images (Fig. S3). When the volume of the decane droplet is sufficiently low to balance the supply of PFD and the expelled daughter droplets, the coaxial structure demonstrates intense random motion in the interior of the Petri dish, as shown in Fig. 2f, reflecting the internal noise of the extended duplex film. On the other hand, when the volume of decane is too high, the supply of PFD from the central PFD droplet and the expulsion of daughter droplets from the duplex film are not balanced, leading to the disappearance of the PFD fringe. The disappearance of this fringe does not occur uniformly along the periphery of the coaxial structure, and this disparity leads to the peripheral flow depicted in Fig. 3d(iii). This flow directs the PFD droplet to the triple-phase contact line to reform the structure shown in Fig. 3d(i). The channel opening and closing behavior discussed for Fig. S2 is considered as a repetition of the event shown in Fig. 3d.

The dynamics of the droplet system are expected to be enhanced by decreasing the decane/water interfacial tension through the addition of a small amount of a surface-active agent, such as 10^−6^ mol/L of Triton-X. It should be noted that trace contaminants in decane can have the same effect^50^. The decrease in the decane/water interfacial tension leads to the spreading of the decane droplet. Furthermore, the spreading of the decane droplet implies the existence of an encircling decane film (Fig. 4a, top view). In the case of surfactant-containing droplet systems with low droplet volumes (corresponding surfactant-free droplet systems have Janus structures, blue domain in Fig. 2a), the decane and PFD droplets remain adjacent once they have come into contact, as shown in Fig. 4b. PFD then spreads across the decane film forming a PFD/decane duplex film. Owing to the volatility of PFD, the duplex film induces an effective repulsion that prevents direct contact between the PFD and decane droplets, resulting in a small gap between the adjacent droplets (Fig. 4b). In this case, unlike the motion observed in the surfactant-free system, the pair of droplets translates with the decane droplet in front, and the decane droplet occasionally rotates around the PFD droplet. See Supplementary movie 5. The dimer-like structure maintains the flow induced by the spreading of the duplex film, inducing translational motion, similar to the Janus structure of the corresponding surfactant-free droplet system.

**Figure 4:**
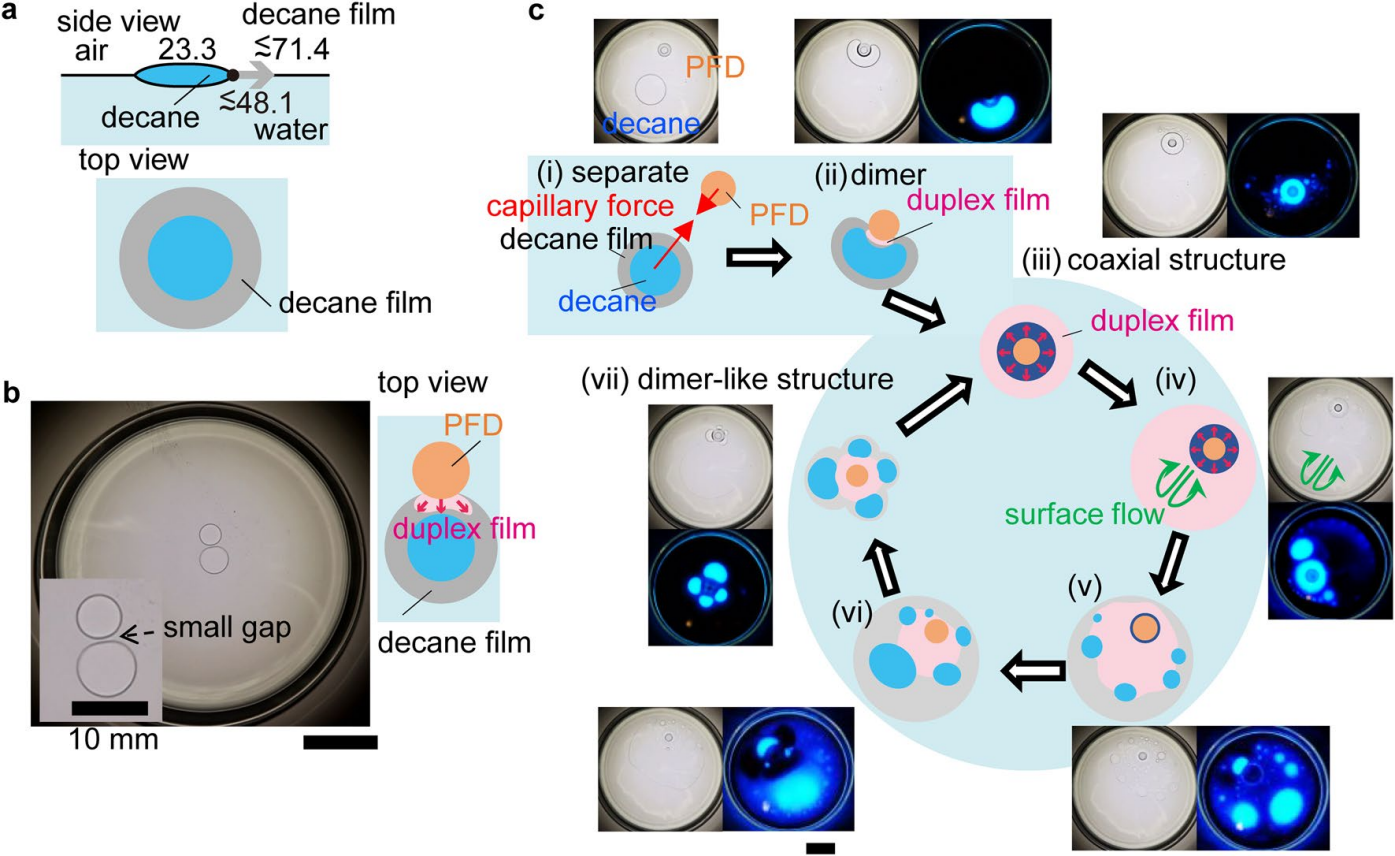
Schematic representation of the cyclic eruptive behavior of droplet systems. (**a**) Side view of a decane droplet showing the effect of the addition of a small amount of surface-active agent, i.e., the decrease in the decane/water interfacial tension which leads to the expansion of the film of decane along the air/water interface. (**b**) Snapshot of the dimer-like structure of a surfactant-containing droplet system with low droplet volumes (10 µL). The top view shows that PFD spreads on the decane film to form the duplex film without the merging of the droplets. See Supplementary movie 5 which demonstrates the translational motion. (**c**) Fluorescence and shadow graph images and illustration of the cyclic eruptive behavior of a surfactant-containing droplet system with high droplet volumes (150 µL). **c(i)**, Temporary dimer structure of the droplet system and **c(ii)**, duplex film formed after capillary interaction causes the PFD and decane droplets to make contact. **c(iii)**, Formation of a coaxial structure. **c(iv)**, The duplex film expands along the air/water interface until the PFD fringe is exhausted. **c(v)–(vi)**, Owing to the high volatility of PFD, the spread decane film forms decane daughter droplets. **c(vii)**, PFD and decane daughter droplets attract each other, forming a structure that is essentially the same as that observed in **c(ii)**. Accordingly, the coaxial structure shown in **c(iii)** is regenerated from **c(vii)**. The process repeats itself. The scale bar corresponds to 20 mm unless stated otherwise.

Unique behavior is exhibited by a surfactant-containing droplet system with high droplet volumes (and a coaxial structure) (Fig. 2a). In this droplet system, the duplex film expands more easily on the already existing decane film at the air/water interface. The active eruption of PFD results in the formation of structures reminiscent of tears of wine^51–54^ outside the coaxial structure. The typical eruptive behavior is shown in Fig. 1b; starting from a coaxial structure, repeated expansion of the duplex film and ejection of daughter droplets (duplex film) occur before the coaxial structure reforms. This cycle can repeat itself a few times (Fig. 4c and Supplementary movie 6). The initial formation of the coaxial structure involves the attraction of the decane and PFD droplets (Fig. 4c(i)), their merging (Fig. 4c(ii)), and subsequent engulfing of the PFD droplet by the decane droplet (Fig. 4c(iii)). This behavior is similar to that of the surfactant-free droplet system. However, the significant expansion of the decane film in this droplet system, revealed by the fluorescent images in Figs. 1b and 4c, differs distinctly from that of the decane film in the surfactant-free droplet system. Strong Marangoni flow in the duplex film is responsible for the spread of the decane film away from the center (Fig. 4c(iv)). A similar Marangoni flow of the duplex film is observed in the surfactant-free droplet system, but the spread of decane is so much greater in the surfactant-containing droplet system that the decane ring around the central PFD droplet is nearly depleted. Although some decane flows back to the central PFD droplet, considerable decane does not return and forms a thick region within the film (Fig. 4c(v)). This is a result of the evaporation of PFD, that causes the dewetting of decane film to form droplets. Decane droplets subsequently grow (Fig. 4c(v),c(vi)) and finally return to the PFD droplet, regenerating the decane ring (Fig. 4c(vii)) and reconstructing the original coaxial structure (Fig. 4c(iii)). Interestingly, the PFD fringe located at the outermost triple-phase contact line also reintegrates into the coaxial structure. The cycle repeats itself several times until the droplets reduce to sufficiently low volumes due to evaporation.

Marangoni flow is caused by the difference in the effective interfacial energies, or the interfacial tensions, between air/PFD/decane/water and air/water and also between air/PFD/decane/water and air/decane/water. We assumed that the duplex film as well as the PFD film that expands at air-decane surface, to be thin enough, generating the effective interfacial tension of film dependent on the thinning process of the evaporating film. It has been argued that such a thin film often assumed to be of the order of a few nm^55^. The effective interfacial tensions of the air/PFD/decane/water and air/PFD/decane systems change as volatile PFD evaporates. Such a change in effective interfacial tension induces the spreading, Marangoni flow, of the PFD/decane duplex film formed at the air/water interface and the PFD film formed at the air/decane interface.

The size-effect of the petri dish was examined by the use of 60 mm and 120 mm diameter dishes. No essential difference in droplet dynamics was observed for the results with 120 mm diameter. However, for 60 mm diameter dish, there was a case that the eruptive behavior shown in Fig. 4 was observed for droplets with 50 µL-each, suggesting that the size effect possibly appears in such a smaller diameter. We performed experiments in which the PFD droplet was fixed by a needle or by its contact to the bottom of petri-dich. Droplets did not show translational motion, but other dynamics was essentially the same.

To conclude, we designed and investigated an artificial droplet system that demonstrates behavior reminiscent of living cells, such as spontaneous pairing and motion, and recurring fission/fusion. When deposited on the surface of water, PFD and decane droplets adopt either a Janus or coaxial structure. In both cases, the droplet system exhibits autonomous motion. The droplet system with a coaxial structure displays periodic eruptive behavior, involving the consecutive destruction and reconstruction of the initial coaxial structure, with a unique spatiotemporal pattern. Instead of a chemical reaction, it is the progressive evaporation of PFD that provides the energy flux necessary to produce the dynamic behavior of the droplet system. Perfluorocarbons are largely immiscible with hydrocarbons, which qualifies them as components of active droplets since they can engender compartmentation. We show here that their high volatility, associated with low van der Waals intermolecular interactions, is also critical for designing active droplets. The unique nonlinear dynamical behaviors of PFD/decane droplets, such as spontaneous erupting, reconstructing, and propelling, are expected to serve as a real-world modeling of prebiotic protocells and proto-organoids that are hypothesized to be membraneless^56, 57^.

## Methods

### Materials

Octadecafluorodecahydronaphthalene (PFD) (purity > 95.0%) was purchased from Tokyo Chemical Industry Co., Ltd. Decane (purity > 99.0%), perylene (purity > 95.0%), and nonionic surfactant (Triton X-100) were purchased from FUJIFILM Wako Chemicals Inc. The water dissolved in decane was removed using a molecular sieve (FUJIFILM Wako Chemicals Inc., 3A1/16) as needed. The other chemicals were used without further purification. Ultra-pure water (Veolia, PURELAB flex 3) was used (resistivity: 18.2 MΩ, surface tension: 72.1 ± 0.2 mN/m at 22 °C).

Procedures. The experimental setup is shown in Fig. 1a. The inner diameter and depth of the glass Petri dish were of 86 and 19 mm, respectively. Water (30 mL) was poured into the Petri dish and a specific volume of PFD or decane (10–200 µL) was carefully deposited on the water surface. After the first droplet settled, a specific volume (10–200 µL) of the second component (decane or PFD) was deposited an appropriate distance (~ 10 mm) from the first droplet. It was found that the order of deposition does not affect the experimental results. A low amount (10^−6^ mol/L) of Triton X-100 was dissolved in the water as necessity requires. Perylene (< 0.1 mg per 10 mL) was dissolved in decane for fluorescence imaging, during which the experiment was performed under UV light (wavelength 254 nm) in a dark room. The movement of the droplets was monitored using a digital camera (Canon, EOS Kiss X9), which enabled the recording of bright field images, fluorescence images, shadowgraphs, and movies.

The interfacial (or surface) tensions were determined by the shape analysis of a pendant drop with a commercial measurement instrument (KSV Instruments, KSV CAM 200). The deviation in the surface tension measurements of water at 22 °C was ± 0.2 mN/m. Considering the fluctuation in room temperature, the deviation in the surface (or interfacial) tension measurements was ± 0.5 mN/m. The deviation in the spreading coefficient of duplex films on water was 0.4 mN/m. The spreading coefficient of PFD across decane on water was 0.6 mN/m. These values are near/or less than the uncertainty of the measurements. However, the agreement between the proposed mechanism and the results of the experiments, especially the observation of Marangoni flows, supports the conclusion that these coefficients are positive.

## Supplementary Information

Supplementary Figures.

Supplementary Movie 1.

Supplementary Movie 2.

Supplementary Movie 3.

Supplementary Movie 4.

Supplementary Movie 5.

Supplementary Movie 6.

Supplementary Legends.

## Data Availability

Data supporting the findings reported in this manuscript are available from the corresponding authors upon reasonable request.

## Code availability

Computer code used to analyze the images is based on Image J^58^ and is available from the corresponding authors upon reasonable request.
