# Supplementary material for: Droplet duos on water display pairing, autonomous motion, and periodic eruption
Source: Sci Rep. 2023 Jul 31;13:12377. doi: 10.1038/s41598-023-39094-6 (PMC10390526; doi:10.1038/s41598-023-39094-6)
Supplement: Supplementary file 1 — Supplementary Figures. [file 41598_2023_39094_MOESM1_ESM.pptx]

## Slide 1
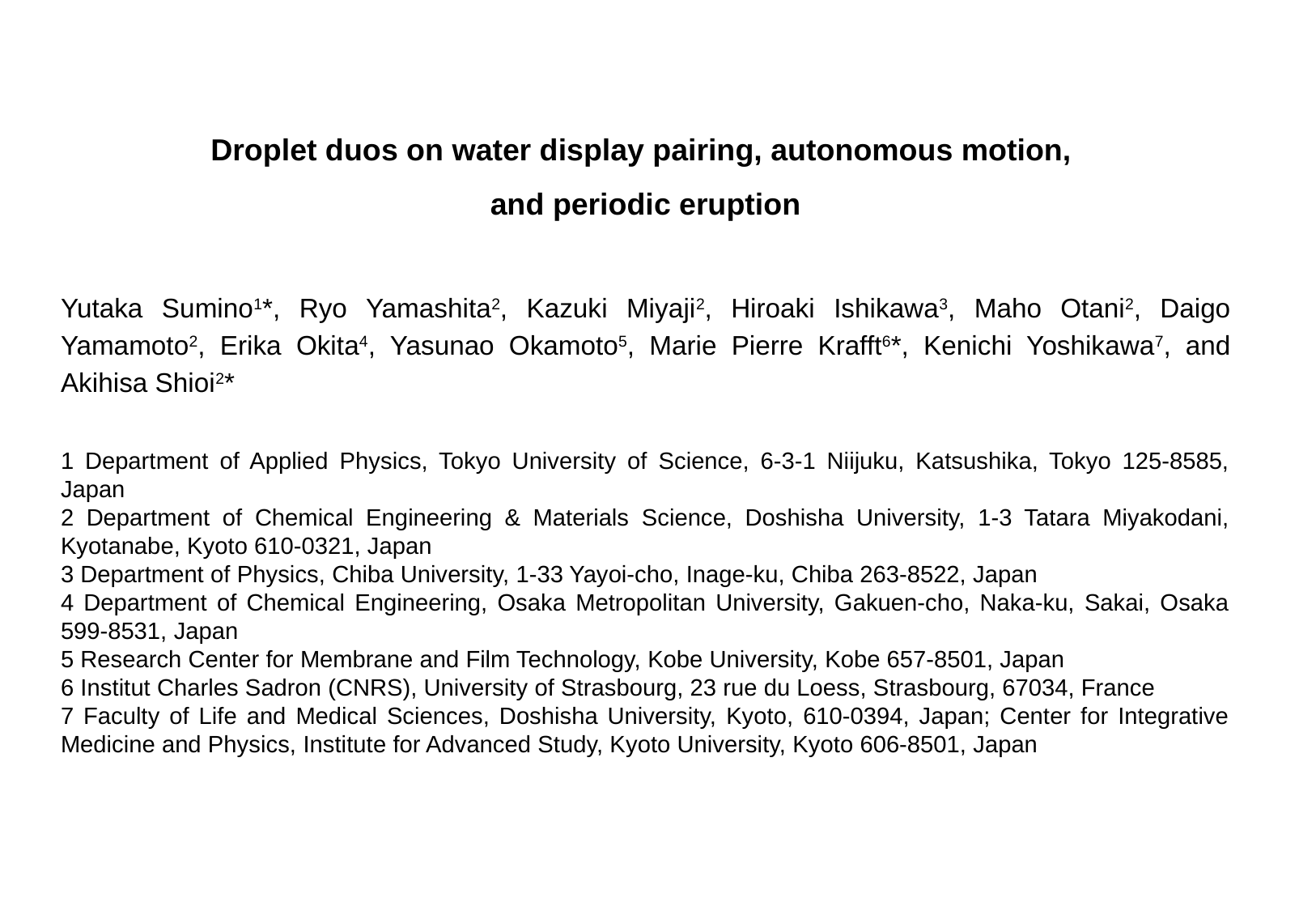

Droplet duos on water display pairing, autonomous motion,
and periodic eruption
Yutaka Sumino1*, Ryo Yamashita2, Kazuki Miyaji2, Hiroaki Ishikawa3, Maho Otani2, Daigo Yamamoto2, Erika Okita4, Yasunao Okamoto5, Marie Pierre Krafft6*, Kenichi Yoshikawa7, and Akihisa Shioi2*
1 Department of Applied Physics, Tokyo University of Science, 6-3-1 Niijuku, Katsushika, Tokyo 125-8585, Japan
2 Department of Chemical Engineering & Materials Science, Doshisha University, 1-3 Tatara Miyakodani, Kyotanabe, Kyoto 610-0321, Japan
3 Department of Physics, Chiba University, 1-33 Yayoi-cho, Inage-ku, Chiba 263-8522, Japan
4 Department of Chemical Engineering, Osaka Metropolitan University, Gakuen-cho, Naka-ku, Sakai, Osaka 599-8531, Japan
5 Research Center for Membrane and Film Technology, Kobe University, Kobe 657-8501, Japan
6 Institut Charles Sadron (CNRS), University of Strasbourg, 23 rue du Loess, Strasbourg, 67034, France
7 Faculty of Life and Medical Sciences, Doshisha University, Kyoto, 610-0394, Japan; Center for Integrative Medicine and Physics, Institute for Advanced Study, Kyoto University, Kyoto 606-8501, Japan

## Slide 2
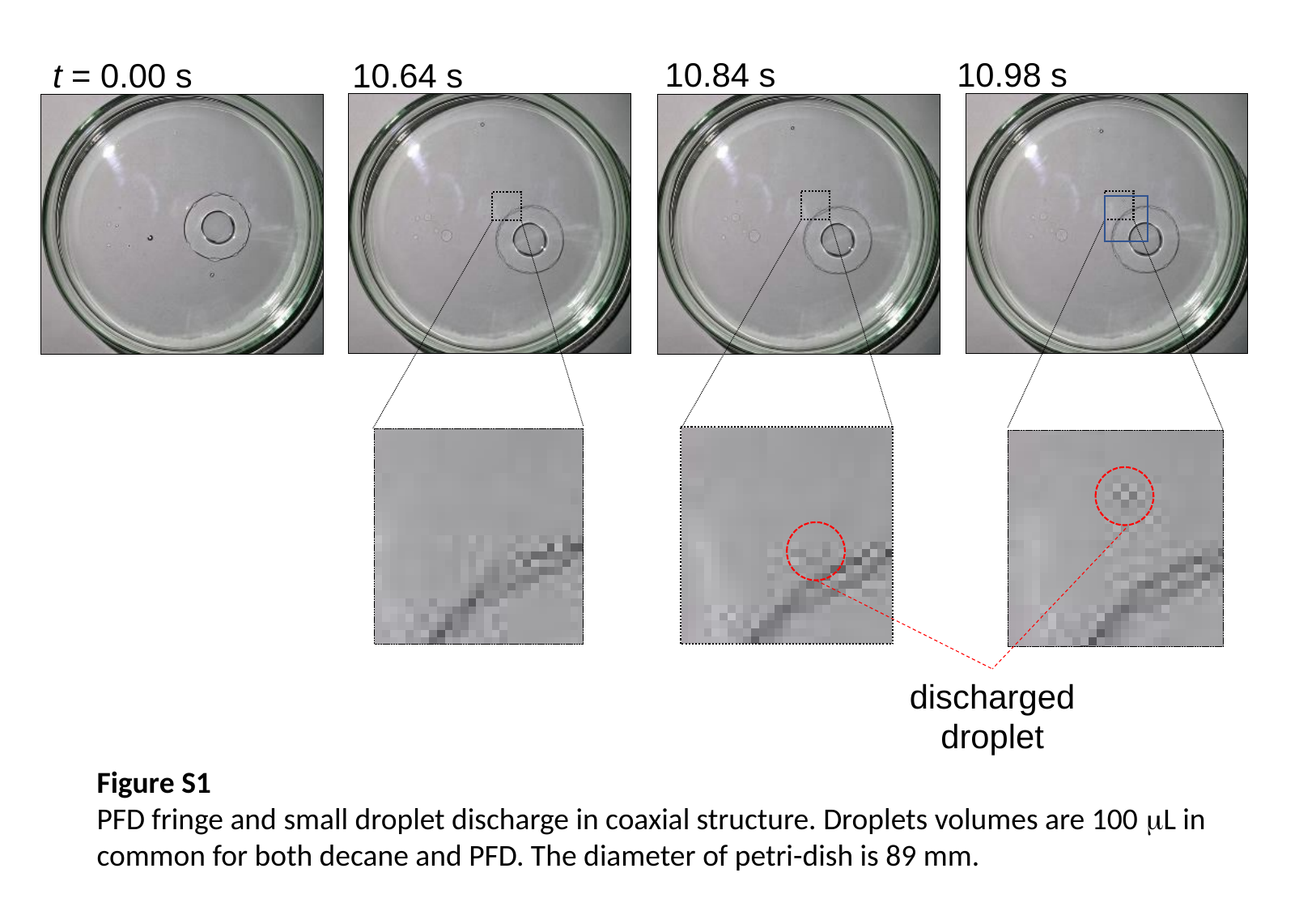

10.98 s
10.84 s
t = 0.00 s
10.64 s
discharged droplet
Figure S1
PFD fringe and small droplet discharge in coaxial structure. Droplets volumes are 100 μL in common for both decane and PFD. The diameter of petri-dish is 89 mm.

## Slide 3
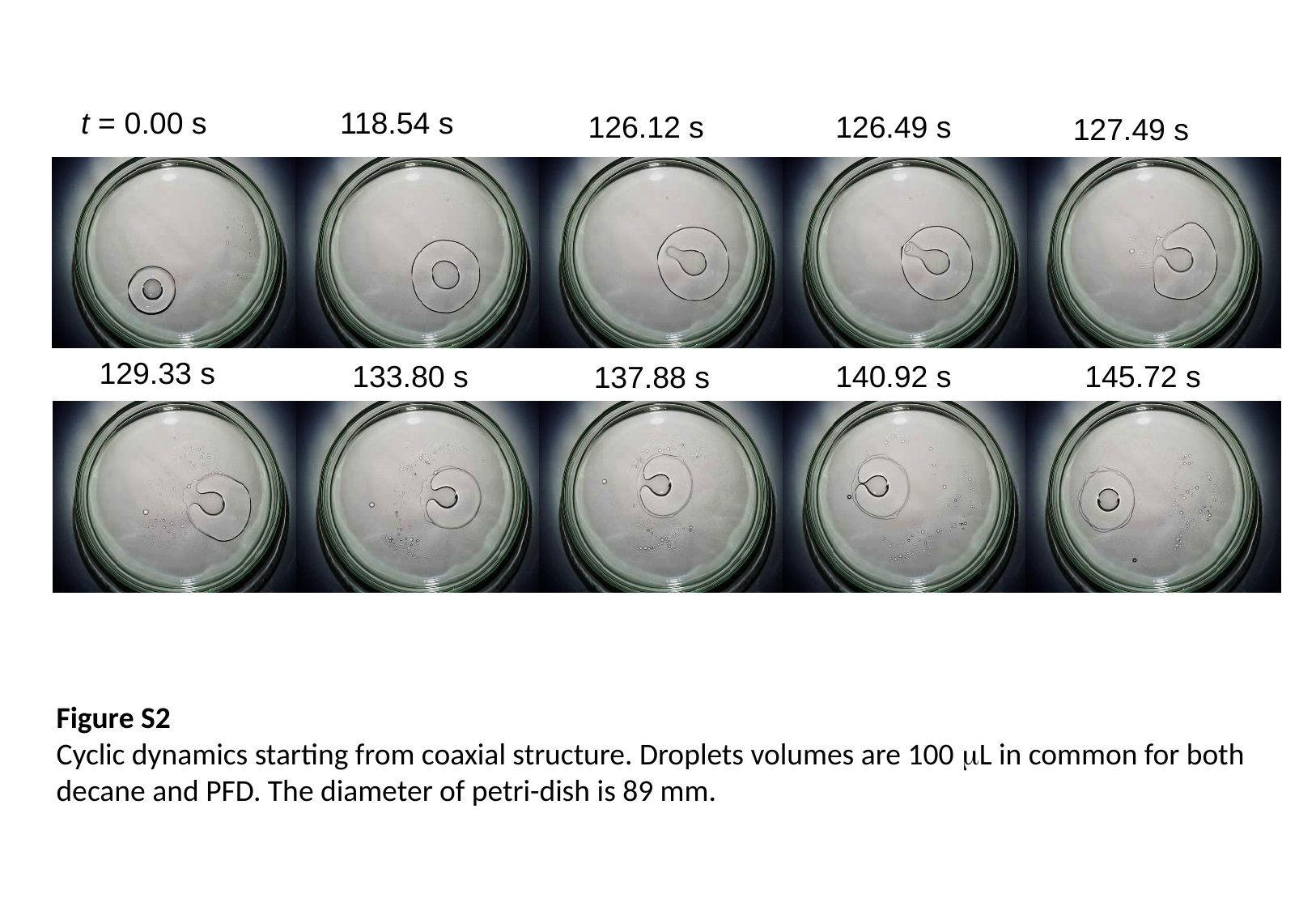

t = 0.00 s
118.54 s
126.12 s
126.49 s
127.49 s
129.33 s
133.80 s
140.92 s
145.72 s
137.88 s
Figure S2
Cyclic dynamics starting from coaxial structure. Droplets volumes are 100 μL in common for both decane and PFD. The diameter of petri-dish is 89 mm.

## Slide 4
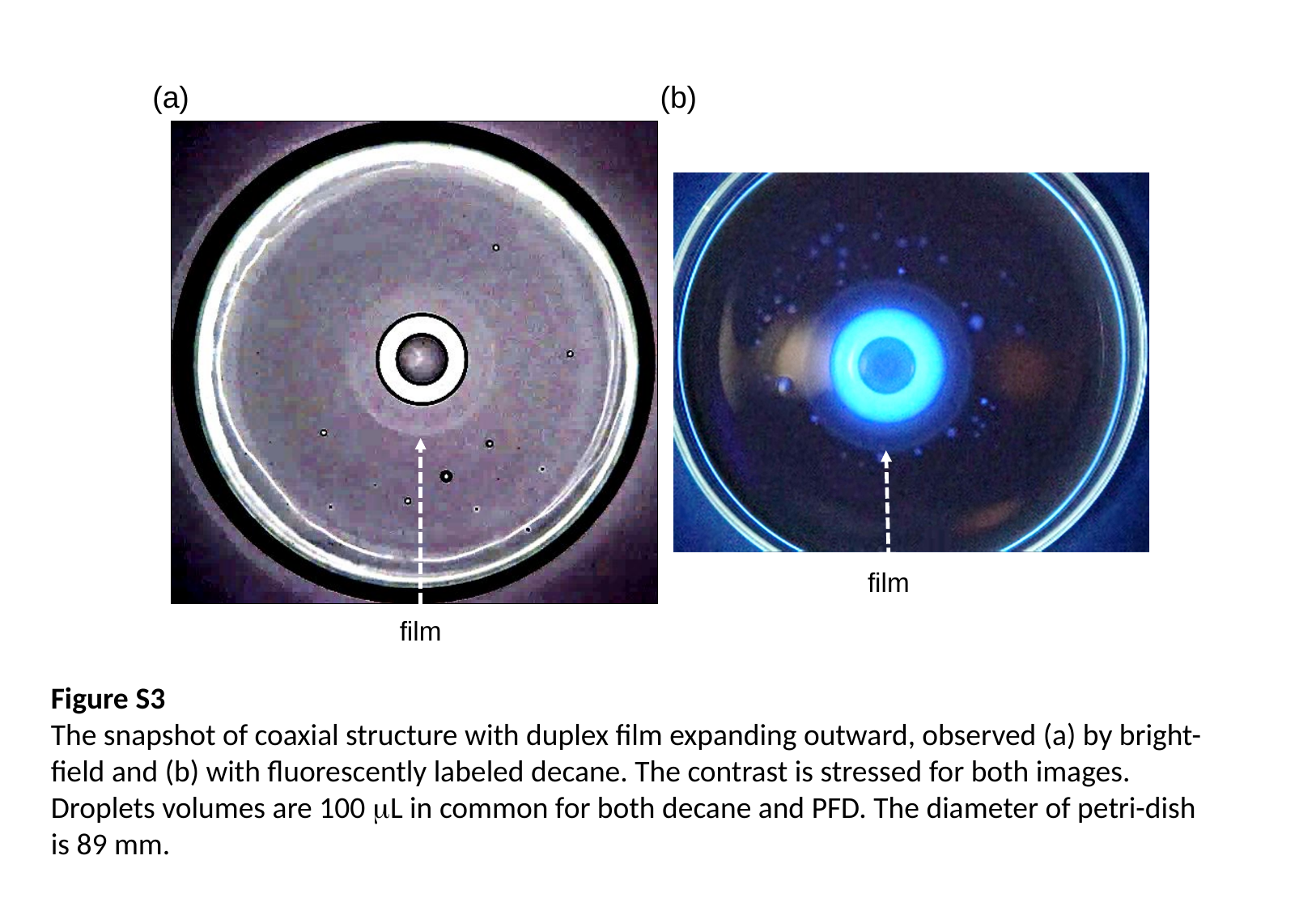

(a)
(b)
film
film
Figure S3
The snapshot of coaxial structure with duplex film expanding outward, observed (a) by bright-field and (b) with fluorescently labeled decane. The contrast is stressed for both images. Droplets volumes are 100 μL in common for both decane and PFD. The diameter of petri-dish is 89 mm.
