# Supplementary material for: Droplet duos on water display pairing, autonomous motion, and periodic eruption
Source: Sci Rep. 2023 Jul 31;13:12377. doi: 10.1038/s41598-023-39094-6 (PMC10390526; doi:10.1038/s41598-023-39094-6)
Supplement: Supplementary file 8 — Supplementary Legends. [file 41598_2023_39094_MOESM8_ESM.pdf]

Supplementary movie 1

Movie of the periodic eruptive behavior of a droplet system featuring PFD and fluorescently labelled decane (the volume of each droplet is 150 µL), reminiscent of a volcanic eruption. The video is sped up 50 times.

Supplementary movie 2

Movie (shadow graph) of decane and PFD droplets (10 µL each) forming a Janus structure. The video is sped up 10 times.

Supplementary movie 3

Movie (shadow graph) of decane and PFD droplets (100 µL each) forming a coaxial structure. The PFD droplet at the center of the structure is completely encircled by the decane droplet. The video is sped up 10 times.

Supplementary movie 4

Visualization, using sprinkled carbon powder, of the Marangoni flow generated by a PFD droplet deposited on decane. Actual speed.

Supplementary movie 5

Movie (shadow graph) of decane and PFD droplets (10 µL each) forming a dimer-like structure on water modified with a surfactant. The dimer-like structure translates with decane in front, which, subsequently, rotates around the PFD droplet. The video is sped up 10 times.

Supplementary movie 6

Movie (shadow graph) of the periodic eruptive behavior of a surfactant-containing droplet system (the volumes of the decane and PFD droplets are 100 μL) reminiscent of a volcanic eruption. Although the large PFD droplet sometimes touches the bottom of the Petri dish, this contact, distinguishable as a center circle, does not affect the dynamic behavior of the system. The video is sped up 50 times.
